# Inclisiran administration potently and durably lowers LDL-C over an extended-term follow-up: the ORION-8 trial

**DOI:** 10.1093/cvr/cvae109

**Published:** 2024-05-16

**Authors:** R Scott Wright, Frederick J Raal, Wolfgang Koenig, Ulf Landmesser, Lawrence A Leiter, Sheikh Vikarunnessa, Anastasia Lesogor, Pierre Maheux, Zsolt Talloczy, Xiao Zang, Gregory G Schwartz, Kausik K Ray

**Affiliations:** Division of Preventive Cardiology, Department of Cardiology, Mayo Clinic, Rochester, MN 55905, USA; Department of Medicine, Faculty of Health Sciences, University of the Witwatersrand, Johannesburg, South Africa; German Heart Centre, DZHK (German Centre for Cardiovascular Research), Partner Site Munich Heart Alliance, Technical University of Munich, Munich, Germany; Institute of Epidemiology and Medical Biometry, University of Ulm, Ulm, Germany; Department of Cardiology, Angiology and Intensive Care Medicine, Deutsches Herzzentrum der Charité, Charité-Universitätsmedizin Berlin, Berlin, Germany; Friede Springer Cardiovascular Prevention Center at Charité, DZHK, Partner Site Berlin, Berlin Institute of Health, Berlin, Germany; St Michael's Hospital, Li Ka Shing Knowledge Institute, University of Toronto, Toronto, ON, Canada; Novartis Pharmaceuticals Corp., East Hanover, NJ, USA; Novartis Pharma AG, Basel, Switzerland; Novartis Pharma AG, Basel, Switzerland; Novartis Pharmaceuticals Corp., East Hanover, NJ, USA; Novartis Pharmaceuticals Corp., East Hanover, NJ, USA; Division of Cardiology, University of Colorado School of Medicine, Aurora, Colorado, USA; Department of Primary Care and Public Health, Imperial Centre for Cardiovascular Disease Prevention, Imperial College, London, United Kingdom

**Keywords:** Inclisiran, Atherosclerotic cardiovascular disease, Low-density lipoprotein cholesterol, Proprotein convertase subtilisin/kexin type 9, Long-term exposure

## Abstract

**Aims:**

Data describing the long-term efficacy and tolerability of inclisiran are limited. This was explored in ORION-8, an open-label extension of preceding Phase 2 and Phase 3 placebo-controlled and open-label extension trials.

**Methods and results:**

Following completion of the parent trial, adult patients with atherosclerotic cardiovascular disease (ASCVD), ASCVD risk equivalent, or heterozygous familial hypercholesterolaemia received open-label inclisiran twice yearly (after initial and 3-month doses) until Day 990, followed by an end-of-study visit at Day 1080 or ≥ 90 days after the last dose. The study endpoints included the proportion of patients achieving pre-specified low-density lipoprotein cholesterol (LDL-C) goals [ASCVD: < 1.8 mmol/L (< 70 mg/dL); ASCVD risk equivalent: < 2.6 mmol/L (< 100 mg/dL)], percentage and absolute changes in LDL-C at end-of-study, and safety of inclisiran. Of 3274 patients, 2446 (74.7%) were followed until end-of-study. Mean age was 64.9 ± 9.9 years, 82.7% (*n* = 2709) had ASCVD, and mean baseline LDL-C was 2.9 ± 1.2 mmol/L. Mean cumulative exposure to inclisiran (including parent trials) was 3.7 years; maximum exposure was 6.8 years. With inclisiran, 78.4% [95% confidence interval (CI): 76.8, 80.0] of patients achieved pre-specified LDL-C goals and mean percentage change in LDL-C was −49.4% (95% CI: −50.4, −48.3). No attenuation of LDL-C lowering over time was observed. Treatment-emergent adverse events at injection site (all mild/moderate) occurred in 5.9% of the patients. Inclisiran-associated anti-drug antibodies were infrequent (5.5%) and had no impact on the efficacy or safety of inclisiran. No new safety signals were identified.

**Conclusion:**

In the largest and longest follow-up to date with >12 000 patient-years exposure, inclisiran demonstrated consistent and effective LDL-C lowering with a favourable long-term safety and tolerability profile.

**Trial Registration number:**

ClinicalTrials.gov identifier: NCT03814187

## Graphical Abstract

**Graphical Abstract:**
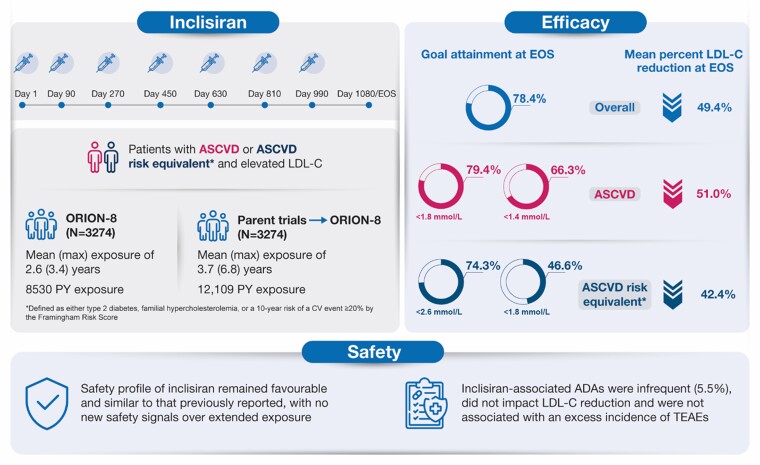
Long-term efficacy and safety of inclisiran. ADA, anti-drug antibody; ASCVD, atherosclerotic cardiovascular disease; EOS, end-of-study; LDL-C, low-density lipoprotein cholesterol; PY, patient-years; TEAE, treatment-emergent adverse event.

**Time of primary review: 28 days**

**See the editorial comment for this article ‘ORION-8: one step closer to understanding the safety and efficacy of inclisiran’, by L. Tokgözoğlu and G.D. Norata, https://doi.org/10.1093/cvr/cvae166.**

## 1. Introduction

Inclisiran is a first-in-class small interfering ribonucleic acid (siRNA) administered twice yearly (after the initial and 3-month doses) via subcutaneous injection by a healthcare provider. Inclisiran targets hepatic proprotein convertase subtilisin/kexin type 9 (PCSK9) mRNA to suppress protein translation in the liver, thereby causing substantial reduction in levels of low-density lipoprotein cholesterol (LDL-C) and other atherogenic lipoproteins.^1–3^

Pivotal data on the lipid-lowering efficacy of inclisiran were established from three placebo-controlled Phase-3 trials, namely ORION-9, ORION-10, and ORION-11, together comprising 3660 patients with elevated LDL-C levels despite optimized oral lipid-lowering therapy (LLT).^4,5^ A pooled analysis of these trials demonstrated that inclisiran reduced LDL-C by ∼51% compared with placebo over an 18-month period with a similar frequency of treatment-emergent adverse events (TEAEs) in both groups.^2^ All placebo-controlled studies completed to date with inclisiran have examined its lipid-lowering efficacy for up to 18 months, with no patient receiving more than four injections of inclisiran.^4,5^ The lipid-lowering efficacy of inclisiran was evaluated in the open-label ORION-3 trial (an extension of the Phase 2 ORION-1 trial) demonstrating a time-averaged mean percentage LDL-C reduction of 44.2% with twice-yearly 300-mg inclisiran sodium (equivalent to 284-mg inclisiran) over 4 years; however, only 382 patients were included in this study, each of whom received up to nine injections of inclisiran.^6^

Long-term data on the durability of LDL-C lowering by inclisiran were evaluated in the ORION-8 trial (NCT03814187), which analysed 3274 participants from four previous inclisiran trials (ORION-9, ORION-10, ORION-11, and ORION-3) for up to 6.8 years of follow-up. A relevant clinical question within the healthcare community is whether the earlier observed data on LDL-C lowering by inclisiran remained consistent with previous reports and was sustained over a longer period of follow-up with twice-yearly dosing. We have previously reported on the safety of a longer-term follow-up period (up to 6 years) representing ∼10 000 patient-years of exposure to inclisrian.^7^ The current report primarily focuses on the consistent efficacy of inclisiran in the largest and longest follow-up to date with > 12 000 patient-years.

Global clinical guidelines have evolved to recommend progressively lower LDL-C goals, particularly for patients at the highest risk of atherosclerotic cardiovascular disease (ASCVD). Recent data highlight the benefit of extended time in range for very-high-risk patients with ASCVD who achieved very low [1.4 mmol/L (< 55 mg/dL)] and low [1.8 mmol/L (< 70 mg/dL)] LDL-C thresholds. Furthermore, it is evident from prior work, including our own, that statin therapy alone or in combination with ezetimibe is often not enough to achieve very low LDL-C thresholds.^8,9^ The addition of non-statin LLT to statins not only results in further LDL-C lowering but also reduces the risk of ASCVD events.^8–10^ ORION-8 evaluates whether the addition of twice-yearly dosage of inclisiran to LLT facilitates consistent LDL-C goal attainment throughout the treatment duration. Here, we report the final results from ORION-8, an open-label extension trial of ORION-9, ORION-10, ORION-11, and ORION-3, the largest and longest assessment to date of the efficacy and tolerability of inclisiran.

## 2. Methods

### 2.1 Study design and participants

**Figure 1:**
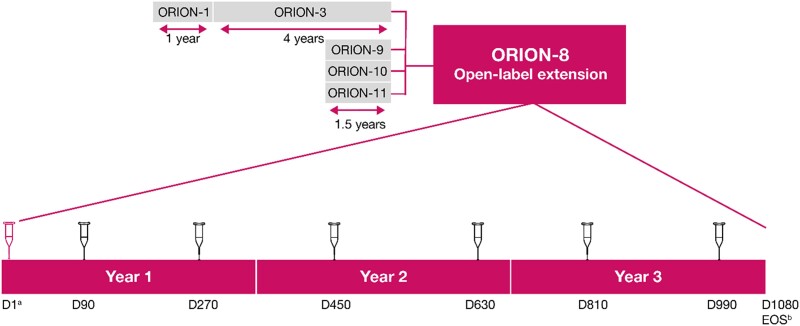
Study design. ^a^Patients from ORION-3 did not receive any drug administration on Day 1. Only patients on placebo in ORION-9, ORION-10, and ORION-11 received an active inclisiran injection at Day 1, while patients who received inclisiran in those trials received blinded placebo at this visit; ^b^EOS is either day 1080 or ≥ 90 days following the last dose of inclisiran. D, Day; EOS, end-of-study.

ORION-8 was a multicentre long-term extension trial of the Phase 2 (ORION-3) and Phase 3 (ORION-9, ORION-10, and ORION-11) lipid-lowering trials (*Figure 1*), conducted across 268 centres in 13 countries in Europe, North America, and Africa. Overall, 3275 patients with ASCVD (coronary heart disease, cerebrovascular disease, or peripheral arterial disease) or ASCVD risk equivalent (defined as type 2 diabetes, familial hypercholesterolaemia, or a 10-year risk of a cardiovascular event ≥ 20% by the Framingham Risk Score^11^), or heterozygous familial hypercholesterolaemia and elevated LDL-C were included, corresponding to 81.0% of the combined parent trial populations.

Adult male and female participants (≥18 years) were eligible for participation in ORION-8 if they completed one of the parent trials (ORION-3, ORION-9, ORION-10, or ORION-11) with no intent to alter the background LLTs used during the parent trial and were willing and able to give informed consent. The exclusion criteria for ORION-8 are provided in the Supplementary material online, *Supplementary Material*. ORION-9, ORION-10, and ORION-11 were 18-month placebo-controlled trials evaluating the lipid-lowering efficacy of inclisiran in patients with heterozygous familial hypercholesterolaemia (ORION-9),^11^ ASCVD (ORION-10, ORION-11), and ASCVD risk equivalent conditions (ORION-11).^5^ ORION-3 was an extension trial for patients from ORION-1 and included patients with ASCVD and ASCVD-risk equivalent.

Day 1 of ORION-8 was defined as the date of the end-of-study visit for the parent trial. Patients who had received blinded placebo in ORION-9, ORION-10, or ORION-11 received blinded 300-mg inclisiran sodium on Day 1 and open-label inclisiran on Day 90 of ORION-8. Patients who had received blinded inclisiran in parent trials received blinded placebo on Day 1 and open-label inclisiran on Day 90 of ORION-8. Patients who rolled over from the ORION-3 did not receive any study medication on Day 1 and received open-label 300-mg inclisiran sodium on Day 90 of ORION-8. After Day 90, all patients returned for dosing visits every 180 days through Day 990. In this manner, all patients in ORION-8 followed a uniform initiation and maintenance dosing schedule for inclisiran. The end-of-study visit for ORION-8 patients originating in ORION-9, ORION-10, or ORION-11 was at Day 1080 or ≥ 90 days after the last dose of inclisiran.

Patients originating from ORION-3 were only followed up in ORION-8 for a shorter duration of a maximum of 1.7 years. The reason for the shorter duration of follow-up was a mutual decision between the Sponsor and regulatory authorities that sufficient long-term safety data would be gathered from ORION-8 participants originating from ORION-9, ORION-10, and ORION-11 trials.

### 2.2 Study endpoints

#### 2.2.1 Efficacy assessments

The primary efficacy endpoint evaluated the proportion of patients who attained pre-specified lipid goals for their level of ASCVD risk at end-of-study, i.e. < 1.8 mmol/L (< 70 mg/dL), and ASCVD risk equivalent, i.e. 2.6 mmol/L (< 100 mg/dL). The selected LDL-C goals were accepted in patients with/without known ASCVD at the time of designing this open-label extension.^10,12^ However, to align with revised LDL-C goals in updated guidelines since this study was designed, goal attainment using the threshold of LDL-C < 1.4 mmol/L (< 55 mg/dL) for ASCVD and < 1.8 mmol/L (< 70 mg/dL) for ASCVD risk equivalent populations was also examined *a posteriori*.^8^ The primary safety endpoint was the evaluation of the safety and tolerability profile of long-term use of inclisiran.

The secondary endpoints evaluated the percentage and absolute changes in LDL-C and other lipids and lipoproteins from baseline (defined as baseline in the parent trial) to end-of-study. Fasted blood samples were drawn at each visit for analysis of LDL-C, total cholesterol, triglycerides, and high-density lipoprotein cholesterol (HDL-C).

#### 2.2.2 Exploratory analysis

An exploratory efficacy analysis was conducted among participants rolled over from ORION-9, ORION-10, and ORION-11 (91% of the aggregate ORION-8 cohort) to determine whether patients treated with inclisiran in the placebo-controlled Phase 3 studies and continuing into ORION-8 had sustained LDL-C reduction across both the parent and extension trials for up to 4.5 years, and whether the LDL-C levels achieved in ORION-8 in those patients were similar to the levels achieved in ORION-8 on inclisiran in patients treated with placebo in the parent trials. This exploratory analysis also examined whether the 1.5-year randomization to placebo prior to initiating inclisiran would result in a different accrual of non-adjudicated major adverse cardiovascular events (MACE) over the entire duration of the parent and extension trials, compared with patients treated with inclisiran from the start of the placebo-controlled parent trials.

#### 2.2.3 Safety assessments

We previously reported a comprehensive safety update on longer-term inclisiran use.^7^ This paper briefly adds to these data and examines whether antidrug antibody (ADA) formation impacted the efficacy and tolerability of long-term inclisiran administration. The Medical Dictionary for Regulatory Activities version 25.1 was used for coding adverse events (see Supplementary material online, *Table S1*). An adverse event [classified by preferred term (PT)] was counted as a TEAE if it was either not present at Day 1 of the study or present at Day 1 of the study but increased in severity during the treatment period. The proportion of patients that reported TEAEs for each PT was tabulated by system-organ class and/or maximum severity. A short set of treatment-emergent safety topics of interest were also examined and included TEAEs at the injection site, hepatic events, new-onset or worsening of diabetes, and MACE-related safety events. If more than one event occurred with the same PT for the same patient, the patient was counted only once for that PT, using the most severe occurrence for the summary by severity.

Blood samples for detection of ADAs were drawn prior to injection and collected at every visit until end-of-study. Persistent and transient ADAs were defined as previously reported.^7^ The percentage change in LDL-C from baseline to end-of-study was summarized for ADA-positive and ADA-negative patients. Safety endpoints in relation to ADA status were presented as exposure-adjusted incidence rates to compensate for different treatment durations.

### 2.3 Statistical analysis

All study data were summarized using descriptive statistics for continuous variables, including number of patients (*n*), mean, standard deviation (SD), median, quartiles (Q1 and Q3), and minimum and maximum values. The analysis of categorical variables included frequency and percentages. Confidence interval (CI) for the mean was based on *t*-test, and for the percentage was calculated using Blaker’s method. The statistical analysis was non-comparative and sample size considerations did not apply. Patient demographics, baseline characteristics, and baseline safety laboratory parameters were representative of the ORION-8 baseline. Medical history and efficacy parameter baseline data were from the ORION-1, ORION-9, ORION-10, and ORION-11 parent trials.

For the exploratory analysis of LDL-C lowering across the parent and extension trials, a mixed model for repeated measures was used to derive the least squares mean percentage change of LDL-C at each visit. The model included fixed effects for treatment, visit, baseline value, parent study, and interaction between treatment and visit. The hazard ratio for the time-to-first event of MACE-related safety composite endpoint was estimated by the Cox model stratified by the parent trials.

### 2.4 Ethics

ORION-8 was conducted in accordance with the trial protocol, principles of the Declaration of Helsinki, and the International Council for Harmonization: Good Clinical Practice, and was approved by the Institutional Review Board of each facility; the specific names of the review boards and ethics committees are described in Supplementary material online, *Supplementary Material*. Written informed consent was obtained from each patient for this open-label extension trial before enrolment. Information on the Institutional Review Boards and ethics committee for each parent trial of ORION-8 (ORION-1, ORION-9, ORION-10, and ORION-11) is as recently described.^7^

## 3. Results

### 3.1 Demographic and clinical characteristics

**Figure 2:**
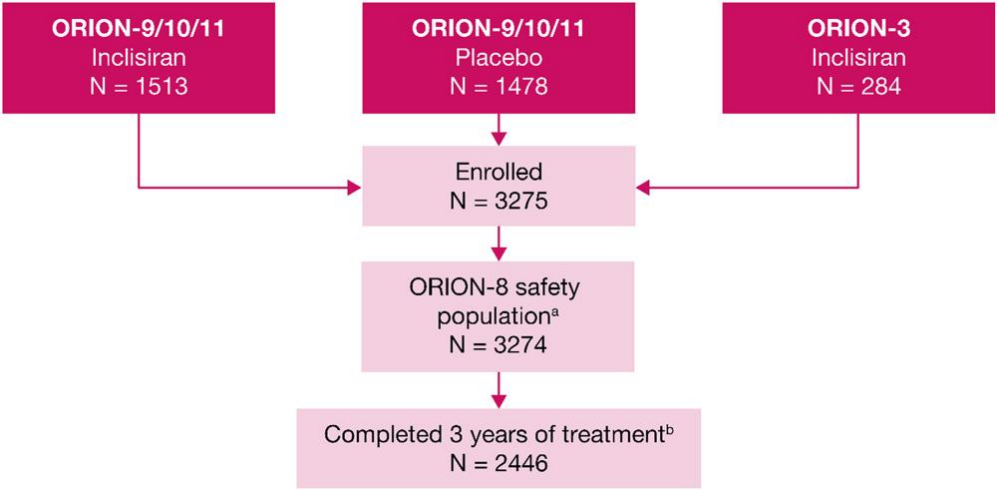
Patient disposition in the ORION-8 trial ^a^One patient from the inclisiran arm of pivotal trials ORION-9, ORION-10, and ORION-11 did not receive any injection in ORION-8. ^b^The primary reasons for discontinuation were ORION-3 rollover patients not offered to complete the full study period (8.3%), death (5.0%), withdrawal of consent (4.8%), lost to follow-up, mostly during the COVID-19 pandemic period (3.1%), other (2.3%), and adverse events (1.4%).

ORION-8 was conducted between 16 April 2019 and 13 February 2023. Of the 3275 patients enrolled in ORION-8, 3274 were included in this analysis. One patient from the inclisiran arm of the pivotal trials (ORION-9, ORION-10, and ORION-11) did not receive any injection in ORION-8 and was excluded (*Figure 2*). The patient demographic and clinical characteristics are summarized in *Table 1*. The mean ± SD age of patients was 64.9 ± 9.9 years, 67.7% (*n* = 2216) of the patients were men, 92.9% (*n* = 3041) were White, and 6.1% (*n* = 199) were Hispanic. The majority (82.7%) of the patients had clinical ASCVD and 17.3% of the patients were classified as ASCVD risk equivalent.

**Table 1:** Baseline demographics and clinical characteristics of the ORION-8 study participants

Characteristic	ORION-8 population, *N* = 3 274
Age (years), mean ± SD	64.9 ± 9.9
Age ≥ 65 years, *n* (%)	1849 (56.5)
Sex, male, *n* (%)	2216 (67.7)
Race, *n* (%)	
White	3041 (92.9)
Black/African American	188 (5.7)
Asian	27 (0.8)
Other (not answered)	18 (0.5)
Ethnicity, Hispanic/Latino, *n* (%)	199 (6.1)
ASCVD status, *n* (%)	
ASCVD	2709 (82.7)
ASCVD risk equivalent	565 (17.3)
LDL-C (mmol/L), mean ± SD	
Overall population	2.9 ± 1.2
ASCVD	2.7 ± 1.0
ASCVD risk equivalent	3.8 ± 1.4
≥ 1 LMT at baseline, *n* (%)	3052 (93.2)
Any statin, *n* (%)	2902 (88.6)
High-intensity statin, *n* (%)	2244 (68.5)
Ezetimibe, *n* (%)	544 (16.6)
Diabetes, *n* (%)	1104 (33.7)
Weight (kg), mean ± SD	88.2 ± 19.13
BMI (kg/m^2^), mean ± SD	30.24 ± 5.76
Smoking status, *n* (%)	
Current smoker	494 (15.1)
Ex-smoker	1480 (45.2)
Never smoked	1291 (39.4)

Baseline refers to ORION-8 baseline, except for medical history and LDL-C from the parent trials.

ASCVD, atherosclerotic cardiovascular disease; BMI, body mass index; LDL-C, low-density lipoprotein cholesterol; LMT, lipid-modifying therapy; *N*, total number of patients; *n*, number of patients in each category; SD, standard deviation.

The mean ± SD baseline LDL-C, derived from the baseline of the parent trials, was 2.9 ± 1.2 mmol/L. Overall, 88.6% (*n* = 2902) of the patients were receiving statins at baseline; 68.5% (*n* = 2244) of the patients were receiving high-intensity statins and 16.6% (*n* = 544) of the patients were receiving ezetimibe. A total of 2446 patients (74.7%) completed the full 3-year period of the trial and 828 (25.3%) patients discontinued. The primary reasons for discontinuation were ORION-3 rollover patients not offered to complete the full study period (8.3%, *n* = 272), death (5.0%, *n* = 165), withdrawal of consent (4.8%, *n* = 157), lost to follow-up, mostly during the COVID-19 pandemic period (3.1%, *n* = 100), other (2.3%, *n* = 76), and adverse events (1.4%, *n* = 45).

### 3.2 Overall exposure to inclisiran

The mean exposure to inclisiran during ORION-8 was 2.6 years, corresponding to 8530 patient-years of exposure. Adding the time exposure in parent trials coupled with additional exposure in ORION-8, the mean cumulative exposure to inclisiran was 3.7 years, corresponding to 12 109 patient-years of exposure. The maximum cumulative exposure to inclisiran was 6.8 years, and a quarter of the patients were exposed to inclisiran for over 4.5 years (see Supplementary material online, *Table S2*).

### 3.3 Study outcomes

#### 3.3.1 Efficacy

**Figure 3:**
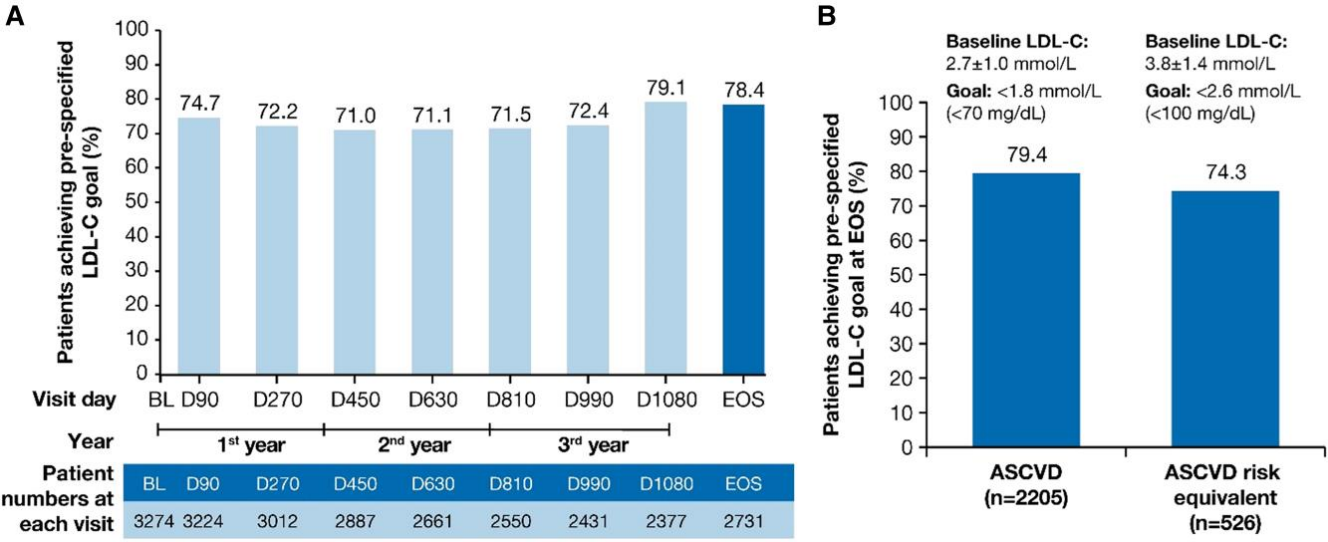
LDL-C goal attainment at EOS. The proportion of patients achieving pre-specified lipid goals at EOS in (*A*) the overall population and (*B*) patients with ASCVD and ASCVD risk equivalent. ASCVD, atherosclerotic cardiovascular disease; BL, baseline; D, Day; EOS, end-of-study; LDL-C, low-density lipoprotein cholesterol; *n*, number of patients at the visit.

The proportion (95% CI) of patients achieving the primary pre-specified LDL-C goal relevant to their clinical category (i.e. ASCVD: < 1.8 mmol/L or < 70 mg/dL; ASCVD risk equivalent: < 2.6 mmol/L or < 100 mg/dL) at end-of-study was 78.4% (95% CI: 76.8–80.0) of 2731 patients. At each visit in ORION-8, more than 70% of the patients achieved the prespecified LDL-C goals (*Figure 3A*).

A total of 79.4% (95% CI: 77.7–81.1) of 2205 patients and 74.3% (95% CI: 70.4–78.0) of 526 patients achieved their pre-specified LDL-C goals at the end-of-study in the ASCVD and the ASCVD risk equivalent populations, respectively (*Figure 3B*). The mean (SD) baseline LDL-C levels were 2.7 ± 1.0 mmol/L and 3.8 ± 1.4 mmol/L in the ASCVD and ASCVD risk equivalent populations, respectively.

On inclisiran, the proportion of patients with ASCVD who achieved LDL-C levels < 1.4 mmol/L (< 55 mg/dL) at end-of-study was 66.3% (95% CI: 64.2–68.2) of 2205 patients; the proportion of patients with ASCVD risk equivalent who achieved LDL-C levels < 1.8 mmol/L (< 70 mg/dL) was 46.6% (95% CI: 42.3–50.9) of 526 patients. The proportion of patients achieving guideline-recommended LDL-C goals of < 1.4, < 1.8 and < 2.6 mmol/L at end-of-study from the different patient populations are presented in Supplementary material online, *Table S3*. In addition, the proportion of patients achieving LDL-C goals at any visit throughout the study post-inclisiran treatment are presented in Supplementary material online, *Figure S1*.

**Figure 4:**
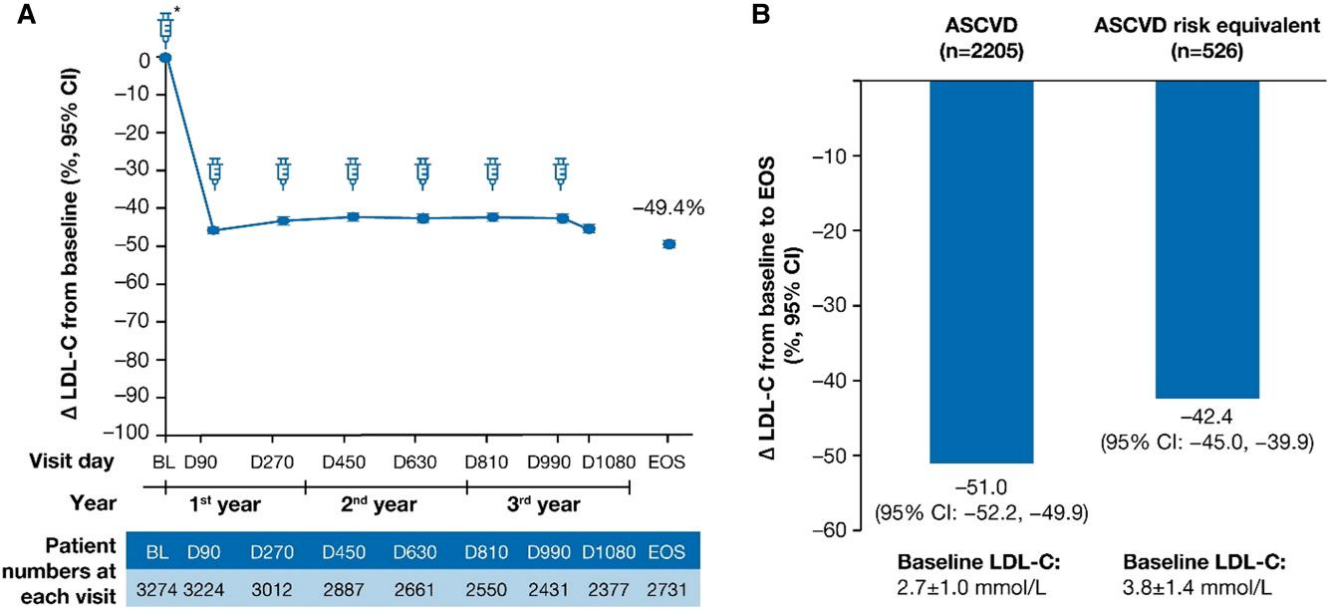
LDL-C percentage change at EOS. The LDL-C percentage change at EOS in (*A*) the overall population and (*B*) patients with ASCVD and ASCVD risk equivalent. Baseline value of LDL-C was taken from the baseline of the parent trials. Δ, mean percentage change; ASCVD, atherosclerotic cardiovascular disease; BL, baseline; CI, confidence interval; D, Day; EOS, end-of-study; LDL-C, low-density lipoprotein cholesterol; *n*, number of patients at the visit.

The percentage change from the parent trial baseline in LDL-C over time is shown in *Figure 4A*. In the overall population, the mean percentage (95% CI) and mean absolute (95% CI) change in LDL-C was −49.4% (−50.4 to −48.3) and −1.5 mmol/L (−1.5 to −1.4) at end-of-study, respectively (see Supplementary material online, *Table S4*). In the ASCVD and the ASCVD risk equivalent populations, the mean percentage (95% CI) change in LDL-C was −51.0% (−52.2 to −49.9) and −42.4% (−45.0 to −39.9) at end-of-study, respectively (*Figure 4B*). The mean percentage and absolute reductions in LDL-C over time are presented in Supplementary material online, *Table S4*.

A total of 11% (*n* = 360) of patients had ≥ 1 new or changed background LLT after Day 1 of ORION-8. A sensitivity analysis was conducted to examine whether these changes to background LLTs may have impacted the efficacy endpoint. In patients without new or changed LLTs from baseline, the percentage (95% CI) of patients achieving pre-specified LDL-C goals, and the mean (95% CI) percentage and absolute changes in LDL-C from baseline at end-of-study were 78.7% (77.0, 80.3), −49.5% (−50.6 to −48.4), and −1.45 mmol/L (−1.49 to −1.41), respectively.

The mean (95% CI) percentage and absolute changes in other lipids and lipoproteins from the parent study baseline at end-of-study are described in *Table 2*. PCSK9, apolipoprotein B, non-HDL-C, and lipoprotein(a) were not evaluated in this study.

**Table 2:** Changes in other lipids and lipoproteins from the parent study baseline at the end-of-study

	Percentage change, mean (95% CI), *n* = 2732	Absolute change, mmol/L, mean (95% CI), *n* = 2732
Total cholesterol	−29.5 (−30.3, −28.8)	−1.5 (−1.6, −1.5)
Triglycerides	−5.1 (−6.8, −3.4)	−0.2 (−0.3, −0.2)
HDL cholesterol	11.0 (10.1, 11.8)	0.1 (0.1, 0.1)

Changes are from the parent trial baseline.

CI, confidence interval; HDL, high-density lipoprotein; *n*, number of patients at end-of-study visit.

#### 3.3.2 Exploratory analyses on patients from ORION-9, ORION-10, and ORION-11 who rolled over to ORION-8

**Figure 5:**
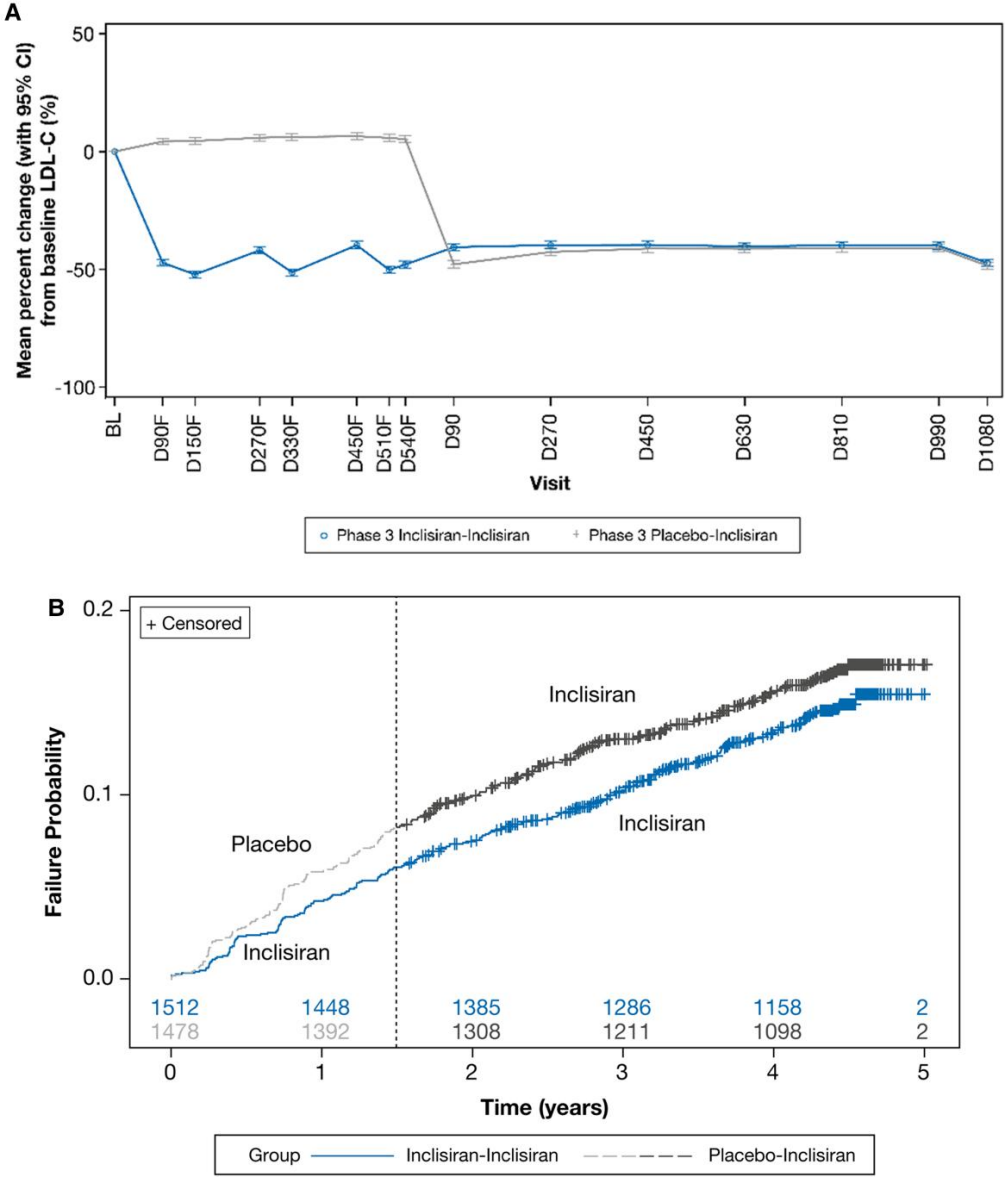
Exploratory analysis of change in LDL-C and MACE-related safety events. The change in (*A*) LDL-C and (*B*) MACE-related safety events. Baseline value of LDL-C was taken from the baseline of the parent trials. To distinguish from visits in ORION-8, the suffix ‘F’ was added to visits in the parent (feeder) trials ORION-9, ORION-10, and ORION-11. The numbers of patients at risk are displayed at the bottom of the Kaplan–Meier plot. The dashed line denotes the transition from the parent trial to ORION-8. BL, baseline; CI, confidence interval; D, Day; LDL-C, low-density lipoprotein cholesterol.

The exploratory analyses demonstrated a sustained and consistent efficacy of inclisiran during both the placebo-controlled period [least square mean (95% CI) percentage change in LDL-C at Day 90 and Day 540 of parent studies was −47.2% (−48.5 to −45.9) and −47.9% (−49.5 to −46.4), respectively] and during the ORION-8 extension period [least square mean (95% CI) percentage change in LDL-C at Day 1080 was −47.2% (−48.8 to −45.6)] (*Figure 5A*). By Day 270 of ORION-8, the percentage change in LDL-C was similar for patients who were treated with inclisiran or placebo during the antecedent placebo-controlled parent trials. Approximately 70% of patients consistently achieved LDL-C goals for their level of ASCVD risk across ORION-9, ORION-10, ORION-11, and ORION-8 for the Phase 3 inclisiran–inclisiran arm (see Supplementary material online, *Table S5*).

Across the parent and extension trials, treatment-emergent MACE-related safety events were reported in 458 patients from the ORION-8 population rolled over from the ORION-9, ORION-10, and ORION-11 trials. A total of 217 (14.4%) of the 1512 patients randomized initially to inclisiran in the parent trials and then continued on inclisiran in ORION-8 experienced MACE-related safety events, whereas 241 (16.3%) of the 1478 patients initially randomized to placebo in the parent trials and then switched to inclisiran in ORION-8 experienced MACE-related safety events [hazard ratio (95% CI): 0.85 (0.71–1.03); *P* = 0.091], suggesting some benefit from longer-term inclisiran exposure or non-significant but modestly higher MACE-related safety events in those initially randomized to placebo. The Kaplan–Meier plot for treatment-emergent MACE-related safety events is shown in *Figure 5B*.

#### 3.3.3 Safety

Approximately 77.8% (*n* = 2548) of the patients had TEAEs (*Table 3*), 30.2% (*n* = 989) had treatment-emergent serious adverse events (TESAEs, *Table 4*), 5.0% (*n* = 165) had fatal TEAEs, and 2.4% (*n* = 80) had TEAEs leading to study drug discontinuation. The most common TEAEs were COVID-19 (13.8%), diabetes mellitus inadequate control (7%), and hypertension (7%) (*Table 3*). The most frequent TESAEs were coronary artery disease (2%), COVID-19 (1.5%), and acute myocardial infarction (1.3%) (*Table 4*). The treatment-emergent safety topics of interest were TEAEs at the injection site (5.9%), hepatic events (5.1%), new-onset or worsening of diabetes (17.8%), and MACE-related safety events (9.2%) (*Table 5*). The TEAEs at the injection site were mostly mild and occasionally moderate (see Supplementary material online, *Table S6*).

**Table 3:** Treatment-emergent adverse events occurring in ≥ 3% of patients for the ORION-8 study participants

Treatment-emergent adverse events	Total, *N* = 3274, *n* (%)
Patients with at least one TEAE	2548 (77.8)
COVID-19	453 (13.8)
Diabetes mellitus inadequate control	229 (7.0)
Hypertension	229 (7.0)
Diabetes mellitus	206 (6.3)
Arthralgia	205 (6.3)
Urinary tract infection	158 (4.8)
Osteoarthritis	149 (4.6)
Back pain	131 (4.0)
Nasopharyngitis	110 (3.4)
Upper respiratory tract infection	110 (3.4)
Atrial fibrillation	100 (3.1)
Coronary artery disease	99 (3.0)

Preferred terms (MedDRA version 25.1) are sorted in descending order of frequency.

COVID-19, coronavirus disease 2019; MedDRA, Medical Dictionary for Regulatory Activities; *N*, total number of patients; *n*, number of patients in each category; TEAE, treatment-emergent adverse event.

**Table 4:** Treatment-emergent serious adverse events occurring in ≥1% of patients for the ORION-8 study participants

Treatment-emergent serious adverse events	Total, *N* = 3274, *n* (%)
Patients with at least one TESAE	989 (30.2)
Coronary artery disease	64 (2.0)
COVID-19	49 (1.5)
Acute myocardial infarction	44 (1.3)
Angina pectoris	43 (1.3)
Osteoarthritis	36 (1.1)
Atrial fibrillation	35 (1.1)
Pneumonia	35 (1.1)
COVID-19 pneumonia	35 (1.1)
Death	33 (1.0)
Myocardial infarction	33 (1.0)

Preferred terms (MedDRA version 25.1) are sorted in descending order of frequency.

COVID-19, coronavirus disease 2019; MedDRA, Medical Dictionary for Regulatory Activities; *N*, total number of patients; *n*, number of patients in each category; TESAE, treatment-emergent serious adverse event.

**Table 5:** Other treatment-emergent safety topics of interest

	Overall population, *N* = 3274			
	TEAEs at injection site, *n* (%)	Hepatic events, *n* (%)	New onset or worsening of diabetes, *n* (%)	MACE-related safety events, *n* (%)
TEAEs	193 (5.9)	166 (5.1)	584 (17.8)	300 (9.2)
TESAEs	0	7 (0.2)	12 (0.4)	228 (7.0)
Fatal TESAEs	0	1 (0.03)	0	86 (2.6)
TEAEs leading to study treatment discontinuation	7 (0.21)	4 (0.1)	0	8 (0.2)

MACE, major adverse cardiovascular event; MedDRA, Medical Dictionary for Regulatory Activities; *N*, total number of patients; *n*, number of patients in each category; TEAE, treatment-emergent adverse event; TEASE, treatment-emergent serious adverse event.

Of 2945 patients from the ORION-8 population with available ADA assessment, excluding patients who received inclisiran in ORION-1, the incidence of inclisiran-associated ADAs was 5.5% (162/2945), with 112 patients (3.8%) who exhibited a transient response and 50 patients (1.7%) who exhibited a persistent response (see Supplementary material online, *Table S7*).

**Figure 6:**
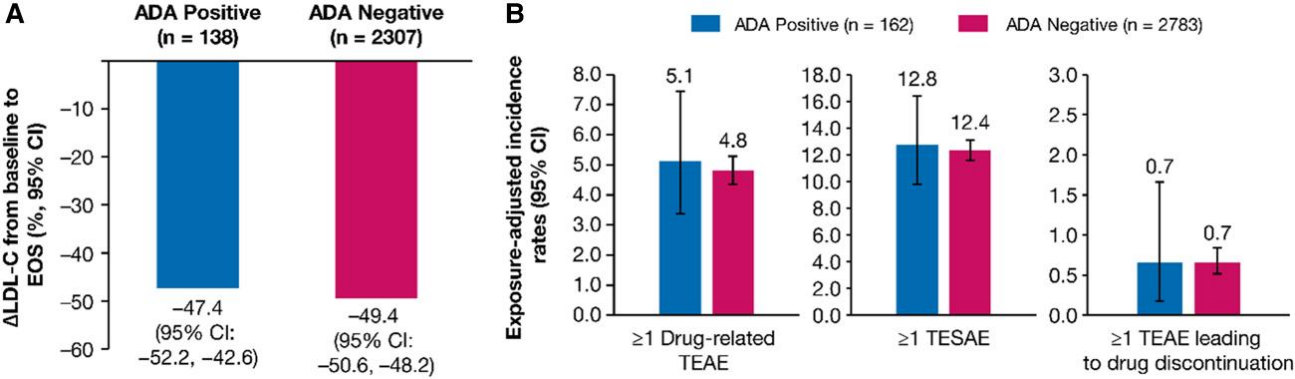
Efficacy and safety by ADA Status. (*A*) Mean percentage change in LDL-C from baseline to EOS, and (*B*) Exposure-adjusted incidence rate (per 100 patient-years) for TEAE and TESAE. The ADA response was determined from the pooled analysis of ORION-3, ORION-9, ORION-10, ORION-11, and ORION-8 ADA data. ORION-1 inclisiran arm patients were excluded from the analysis because the laboratory protocol used in the ORION-1 ADA assessment was different from other studies. The exposure-adjusted incidence rates of TEAEs were calculated since the first inclisiran injection across the parent and extension studies. Δ, mean percentage change; ADA, antidrug antibody; EOS, end of study; LDL-C, low-density lipoprotein cholesterol; TEAE, treatment-emergent adverse event; TESAE, treatment-emergent serious adverse event.

The mean (95% CI) percentage change in LDL-C from baseline to end-of-study in ADA-positive (*n* = 138) and ADA-negative (*n* = 2307) patients was −47.4% (−52.2 to −42.6) and −49.4% (−50.6 to −48.2), respectively, indicating that ADA did not limit treatment efficacy (*Figure 6A*). The mean (95% CI) absolute change in LDL-C from baseline to end-of-study in ADA-positive (*n* = 138) and ADA-negative (*n* = 2307) patients was −1.38 (−1.55, −1.22) and −1.44 (−1.48, −1.40) mmol/L, respectively.

Exposure-adjusted incidence rates (95% CI) per 100 patient-years for ≥ 1 drug-related TEAE [5.1 (3.4–7.5) vs. 4.8 (4.4–5.3)], ≥ 1 treatment-emergent serious adverse event [12.8 (9.8–16.4) vs. 12.4 (11.6–13.2)], and ≥1 TEAE leading to study treatment discontinuation [0.7 (0.2–1.7) vs. 0.7 (0.5–0.8)] were comparable among patients who were ADA-positive (*n* = 162) vs. ADA-negative (*n* = 2783) (*Figure 6B*).

## 4. Discussion

Inclisiran is a first-in-class siRNA therapy with potent LDL-C-lowering properties when tested in placebo-controlled comparisons for up to 18 months. Regulatory approval of inclisiran and wider clinical use necessitated pre-planned efficacy and safety evaluations for an extended treatment period. A previous report supported the safety of inclisiran treatment in 3576 patients for up to 6 years with a mean observation time of 2.8 years. Our prior publication did not report or comment on the efficacy of inclisiran during extended follow-up, with regard to sustained LDL-C lowering, or whether legacy randomization to inclisiran conferred any protection compared with those randomized to placebo.^7^ ORION-8 was a pre-planned analysis of the long-term efficacy of inclisiran treatment in patients enrolled in placebo-controlled Phase 2 and Phase 3 trials, as part of a pre-specified, ongoing open-label extension analysis.

Previously published pooled analysis of the Phase 3 ORION-9, ORION-10, and ORION-11 trials in 1833 patients treated with inclisiran (approximately 2653 patient-years of exposure) demonstrated the effective lowering of LDL-C and the favourable safety profile of twice-yearly inclisiran dosage over an 18-month follow-up period.^2^ Additional data from the Phase 2 ORION-3 trial (approximately 1460 patient-years of exposure including ORION-1) were consistent with these pooled findings and demonstrated sustained LDL-C lowering over a 4-year follow-up period, but this analysis was limited by the small sample size.^6^

Our findings from ORION-8 demonstrated that inclisiran is an effective LLT with sustained and substantial efficacy as an LDL-C-lowering therapy, extending the prior, shorter-term observations from ORION-3, ORION-9, ORION-10, and ORION-11 that comprised 4113 patient-years of inclisiran exposure (ORION-3^6^: 1210 patient-years in inclisiran-only arm in ORION-1 through ORION-3 and 250 patient-years in the switching arm; plus pooled data from ORION-9, ORION-10, and ORION-11 trials^2^: 2653 patient-years). ORION-8 included patients with clinical ASCVD and those in a high-risk primary prevention category over an additional 3-year follow-up period, resulting in 8530 patient-years of inclisiran exposure in ORION-8 and a total of 12 109 patient-years of inclisiran exposure including the parent trials.

In a previously published pooled analysis of ORION-9, ORION-10, and ORION-11, the proportions of patients achieving pre-specified LDL-C goals of < 2.6 mmol/L (< 100 mg/dL) and < 1.8 mmol/L (< 70 mg/dL) at Day 510 were 80.2 and 67.9%, respectively.^2^ In the current analysis, similar LDL-C goal attainment was achieved in 88.6 and 73.1% of patients, respectively, at Day 1080/end-of-study. Furthermore, for ORION-8 patients with ASCVD and categorized as very high risk per the 2019 European Society of Cardiology and European Atherosclerosis Society (ESC/EAS) guidelines,^8^ 66.3% of the patients achieved the LDL-C goal of < 1.4 mmol/L (< 55 mg/dL).

These observations indicate durable fulfilment of guideline-based LDL-C goals in a majority of the patients treated with inclisiran. LDL-C goal attainment at end-of-study was slightly lower in patients with ASCVD risk equivalent than in patients with clinical ASCVD, despite a numerically greater absolute reduction in LDL-C at end-of-study in the former category [−1.7 mmol/L (95% CI: −1.8 to −1.5) vs. −1.4 mmol/L (−1.4 to −1.4)]. This finding is likely due to the fact of higher baseline LDL-C levels in patients with ASCVD risk equivalent conditions than in those with clinical ASCVD (mean baseline LDL-C 3.8 vs. 2.7 mmol/L, *Table 1*). The changes in other lipid/lipoproteins in ORION-8, including total cholesterol, triglycerides, and HDL-C, were also consistent with the prior, shorter-term observations from ORION-9, ORION-10, and ORION-11.^4,5^ In addition, the long-term safety profile of inclisiran remained favourable and similar to that previously reported,^7^ with no new safety signals identified. Similar to prior shorter-term observations,^2,6^ mild-to-moderate TEAEs at the injection site occurred in 5.9% of the patients treated with inclisiran, which resolved without sequelae, did not worsen with long-term treatment, and seldom led to study drug discontinuation. Similar findings have been observed with PCSK9 monoclonal antibodies.^13,14^

There was no evidence that the long-term lipid-lowering efficacy of inclisiran was attenuated by the formation of inclisiran-associated ADAs. Inclisiran-associated ADAs were infrequent (5.5%), did not impact LDL-C reduction, and were not associated with an increased incidence of TEAEs, TESAEs, and TEAEs leading to study drug discontinuation.

Data from the FOURIER-OLE suggested a legacy benefit for those randomized initially to PCSK9 therapy vs. those started on it during the open-label extension period.^15^ Accordingly, we evaluated the potential for such a legacy benefit in our limited data set with the recognition that none of our parent trials were powered to assess cardiovascular outcomes and clinical efficacy cannot be reliably assessed in open-label extension. With these caveats, it appeared that earlier initiation of inclisiran (i.e. assignment to inclisiran vs. placebo in the double-blind treatment period of the parent trials) was associated with a numerically lower incidence of MACE-related safety events. The long-term effect of inclisiran on cardiovascular events is currently under further investigation in large, placebo-controlled trials, ORION-4 (NCT03705234), VICTORION-1 Prevent (NCT05739383), and VICTORION-2 Prevent (NCT05030428).^16–18^

### 4.1 Study limitations

Patients voluntarily entered this open-label extension, and only patients who completed either the Phase 2 open-label extension ORION-3 or one of the three Phase 3 (ORION-9, ORION-10, or ORION-11) trials were eligible for participation. The potential selection bias due to voluntary patient enrolment should be considered when interpreting the results. The absence of a placebo arm during longitudinal extension limits the interpretation of safety, tolerability, and efficacy observations during that period. However, the parent trials had placebo-controlled arms for 18 months and the LDL-C reduction did not substantially differ across all time periods, including placebo-controlled and non-placebo-controlled time periods.

In conclusion, with a cumulative treatment duration of up to 6.8 years, ORION-8 demonstrated that twice-yearly administration of inclisiran provides consistent and effective LDL-C lowering and is well tolerated in patients with high cardiovascular risk, with no new safety signals identified.

**Translational perspective**

ORION-8, a 3-year open-label extension of the placebo-controlled Phase-3 trials ORION-9, ORION-10, ORION-11, and the open-label extension Phase-2 trial, ORION-3, provides the largest and longest follow-up data on inclisiran to date. Twice-yearly administration of inclisiran (after the initial and 3-month doses) demonstrated consistent and effective LDL-C lowering with no new safety signals during an additional mean treatment exposure of 2.6 years beyond the parent trials, with 8530 patient-years of exposure. These findings demonstrate the consistent long-term efficacy and tolerability of inclisiran over a mean cumulative exposure of 3.7 years and maximum exposure of 6.8 years.

## Supplementary Material

cvae109_Supplementary_Data

## Data Availability

Novartis is committed to sharing with qualified external researchers access to patient-level data and supporting clinical documents from eligible studies. These requests are reviewed and approved by an independent review panel on the basis of scientific merit. All data provided are anonymized to respect the privacy of patients who have participated in the trial in line with applicable laws and regulations. This availability of the trial data is according to the criteria and process described on www.clinicalstudydatarequest.com.
