# Supplementary material for: Inclisiran administration potently and durably lowers LDL-C over an extended-term follow-up: the ORION-8 trial
Source: Cardiovasc Res. 2024 May 16;120(12):1400–10. doi: 10.1093/cvr/cvae109 (PMC11481169; doi:10.1093/cvr/cvae109)
Supplement: cvae109_Supplementary_Data [file cvae109_supplementary_data.docx]

# SUPPLEMENTARY MATERIAL

## 1. Study design and participants

*Exclusion criteria*

Patients were excluded from the study if any of the following exclusion criteria applied prior to entry into the study:

1. Any uncontrolled or serious disease, or any medical or surgical condition, that may either interfere with participation in the clinical study and/or put the patient at significant risk (according to investigator’s [or delegate’s] judgment) if he/she participated in the clinical study.
2. An underlying known disease or surgical, physical, or medical condition that, in the opinion of the investigator (or delegate), may interfere with the interpretation of the clinical study results.
3. Severe concomitant non-cardiovascular disease that carries the risk of reducing life expectancy to less than 3 years.
4. Active liver disease, defined as any known current infectious, neoplastic, or metabolic pathology of the liver, or unexplained alanine aminotransferase (ALT) or aspartate aminotransferase (AST) elevation >3× the upper limit of normal (ULN), or total bilirubin (TBIL) elevation >2× ULN at the last recorded visit in the parent study prior to study entry visit.
5. Female participants who are pregnant or nursing or of childbearing potential and unwilling to use at least one method of acceptable effective contraception (eg, oral contraceptives, barrier methods, approved contraceptive implant, long-term injectable contraception, intrauterine device) for the entire duration of the study. Exemptions from this criterion:
   1. Women >2 years post-menopausal (defined as 1 year or longer since last menstrual period) and more than 55 years of age
   2. Post-menopausal women (as defined above) and less than 55 years of age with a negative pregnancy test within 24 hours of enrollment
   3. Women who were surgically sterilized at least 3 months prior to enrollment
6. Planned use of other investigational medicinal products other than inclisiran or devices during the study
7. Any condition that, according to the investigator, could interfere with the conduct of the study, such as but not limited to:
   1. Patients who are unable to communicate or to cooperate with the investigator
   2. Unable to understand the protocol requirements, instructions, and study-related restrictions and the nature, scope, and possible consequences of the study (including patients whose cooperation is doubtful due to drug abuse or alcohol dependency)
   3. Unlikely to comply with the protocol requirements, instructions, and study-related restrictions (eg, uncooperative attitude, inability to return for follow-up visits, and improbability of completing the study)
   4. Had any medical or surgical condition, which in the opinion of the investigator would put the patient at increased risk from participating in the study
   5. Persons directly involved in the conduct of the study

**2. List of Institutional Review Boards and Ethics Committees**

| **Country** | **Institutional Review Board/Ethics Committee Name** | **Type** |
| --- | --- | --- |
| **United States** |  |  |
|  | Advarra, Puyallup, WA | Central |
|  | Atlantic Health System, Morristown, NJ | Local |
|  | Penn State College of Medicine, Hershey, PA | Local |
| **Canada** |  |  |
|  | Institut de recherches cliniques de Montreal, Montreal | Local |
|  | Advarra, Inc, Aurora, Ontario | Central |
|  | Bannatyne Campus Research Ethics Board, Winnipeg, Manitoba | Local |
|  | Health Research Ethics Authority, St. John’s, New Foundland NL | Local |
| **South Africa** |  |  |
|  | Pharma-Ethics Independent Research Ethics Committee, Lyttelton Manor, Gauteng | Central |
|  | Stellenbosch University Health Research Ethics Committee, Cape Town, Western Cape | Local |
| **Netherlands** |  |  |
|  | Central Committee on Research Involving Human Subjects (CCMO), The Hague Zuid-Holland | Central |
| **Spain** |  |  |
|  | CEIm Hospital Clinic de Barcelona, Barcelona, Cataluña | Central |
| **Denmark** |  |  |
|  | The Central Denmark Region Committees on Health Research Ethics, Viborg | Central |
| **Sweden** |  |  |
|  | Etikprövningsmyndigheten, Uppsala | Central |
| **Czech Republic** |  |  |
|  | Eticka komise IKEM a Thomayerovy nemocnice, Praha | Central |
| **Hungary** |  |  |
|  | Medical Research Council Ethics Committee for Clinical Pharmacology, Budapest | Central |
| **United Kingdom** |  |  |
|  | London - Central Research Ethics Committee, Manchester, M1 | Central |
| **Poland** |  |  |
|  | Komisja Bioetyczna przy Dolnoslaskiej Izbie Lerkarskiej, Wroclaw, Dolnoslaskie | Local |
| **Germany** |  |  |
|  | Ethikkommission der Landesärztekammer Hessen, Frankfurt am Main, Hessen | Central |
|  | Ethik-Kommission der Sächsischen Landesärztekammer, Dresden, Sachsen | Local |
|  | Ethik-Kommission der Ärztekammer Westfalen-Lippe, Münster, Nordrhein-Westfalen | Local |
|  | Landesamt für Gesundheit und Soziales Berlin (LaGeSo) Ethik, Berlin | Local |
|  | Ethikkommission der Medizinischen Fakultät Heidelberg, Heidelberg, Baden-Württemberg | Local |
|  | Ethikkommission der Fakultät für Medizin der Technischen Universität München, München, Bayern | Local |
| **Ukraine** |  |  |
|  | Ethics Committee at Medical Centre Subsidiary company «Medical research and practice association Medbud» of Joint Stock Company «Holding Company «Kyivmiskbud», Kyiv, Kyivs'ka Oblast' | Local |
|  | Ethics Committee at Kyiv Railway Clinical Hospital No.2 of the branch of “Healthcare Center” of Joint Stock Company “Ukrainian Railway”, Kyiv, Kyivs'ka Oblast' | Local |
|  | Ethics Committee at SI National Scientific Centre of Radiation Medicine of NAMS of Ukraine, Kiev, Kyivs'ka Oblast’ | Local |
|  | Ethics Committee at Communal nonprofit enterprise ''Cherkasy Regional Hospital of Cherkasy Oblast Council'', Cherkasy, Cherkas'ka Oblast’ | Local |
|  | Ethics Committee at Communal Noncommercial enterprise"Kharkiv City Clinical Hospital #8"of Kharkiv City Council, Kharkiv, Kharkivs'ka Oblast' | Local |
|  | Ethics Committee at Municipal hospital #6 non-profit enterprise«City hospital» Zaporizhzhia City Council, Zaporizhzhia, Zaporiz'ka Oblast' | Local |

# SUPPLEMENTARY TABLES

**Supplementary Table 1. MedDRA-Defined Terms for the Identification of Safety Topics of Interest**

| **Endpoint** | **MedDRA-Defined Terms Using Hierarchical Categories or SMQ** |
| --- | --- |
| Adverse events at the injection site | Injection site reactions (HLT)  Adverse events identified by the investigator as occurring at the injection site |
| Hepatic events | Drug-related hepatic disorders (SMQ, broad and narrow) |
| New onset/worsening of diabetes | Hyperglycaemia/new onset diabetes mellitus (SMQ, narrow)  Diabetic complications (HLGT)  Diabetes mellitus (including subtypes) (HLT)  Carbohydrate tolerance analyses (including diabetes) (HLT), excluding PT “Blood glucose decreased” |
| MACE-related safety | CV death   - Cardiac death   - Fatal SAEs in “Cardiac disorders” SOC   - Fatal SAEs in “General disorder” SOC: PTs of “Death,” “Sudden cardiac death,” “Cardiac death,” “Apparent death” - Fatal stroke   - Central nervous system haemorrhages and cerebrovascular accidents (HLT), fatal events only   Resuscitated cardiac arrest   - Non-fatal adverse events with PT “cardiac arrest”   Non-fatal MI   - MI (SMQ, broad and narrow), non-fatal events only   Non-fatal stroke   - Central nervous system haemorrhages and cerebrovascular accidents (HLT), non-fatal events only |

CV = cardiovascular; HLGT = high-level group term; HLT = high-level term; MACE = major adverse cardiovascular event; MedDRA = Medical Dictionary for Regulatory Activities; MI = myocardial infarction; PT = preferred term; SAE = serious adverse event; SMQ = Standardised MedDRA Query; SOC = system organ class.

**Supplementary Table 2. Duration of Exposure to Inclisiran**

| **Category** | **Inclisiran Exposure in ORION-8 Only N = 3274** | **Cumulative Inclisiran Exposure Including the Parent Trials**  **N = 3274** |
| --- | --- | --- |
| Patient-years of exposure | 8529.9 | 12,109.3 |
| Duration of exposure, years, mean ± SD | 2.6 ± 0.7 | 3.7 ± 1.2 |
| Median, years | 3.0 | 3.3 |
| Q1, Q3, years | 2.82, 3.00 | 2.96, 4.45 |
| Min, Max, years | 0.01, 3.36 | 0.01, 6.84 |
| Number and percentage of patients by duration of exposure, n (%) | | |
| >6 years | - | 209 (6.4) |
| >5 to ≤6 years | - | 4 (0.1) |
| >4 to ≤5 years | - | 1340 (40.9) |
| >3 to ≤4 years | 851 (26.0) | 542 (16.6) |
| >2 to ≤3 years | 1806 (55.2) | 999 (30.5) |
| >1 to ≤2 years | 391 (11.9) | 96 (2.9) |
| ≤1 year | 226 (6.9) | 84 (2.6) |

N = total number of patients; n = number of patients in each category; Q = quartile; SD = standard deviation.

**Supplementary Table 3. Proportion of Patients Achieving** **Guideline-Recommended LDL-C Goals at the End of Study**

| **LDL-C Goal** | **Population** | | |
| --- | --- | --- | --- |
|  | **Overall Safety Population**  **(n = 2731),**  **% (95% CI)** | **ASCVD**  **(n = 2205),**  **% (95% CI)** | **ASCVD Risk Equivalent (n = 526), % (95% CI)** |
| **<1.4 mmol/L**  **(<55 mg/dL)** | 59.4 (57.5-61.2) | 66.3 (64.2-68.2) | 30.6 (26.8-34.7) |
| **<1.8 mmol/L**  **(<70 mg/dL)** | 73.1 (71.4-74.7) | 79.4 (77.7-81.1) | 46.6 (42.3-50.9) |
| **<2.6 mmol/L**  **(<100 mg/dL)** | 88.6 (87.3-89.7) | 92.0 (90.8-93.1) | 74.3 (70.4-78.0) |

CI = confidence interval; LDL-C = low-density lipoprotein cholesterol; n = number of patients at end of study visit.

**Supplementary Table 4: Percentage and Absolute Change in LDL Cholesterol From Baseline by Visit Day**

| **Day** | **n** | **Mean Percentage Change, %**  **(95% CI)** | **Mean Absolute Change,**  **mmol/L**  **(95% CI),** |
| --- | --- | --- | --- |
| **Overall population** | | | |
| Day 90 | 3224 | −45.8 (−46.7 to −44.8) | −1.3 (−1.4 to −1.3) |
| Day 270 | 3012 | −43.3 (−44.4 to −42.2) | −1.3 (−1.3 to −1.2) |
| Day 450 | 2887 | −42.3 (−43.4 to −41.2) | −1.2 (−1.3 to −1.2) |
| Day 630 | 2661 | −42.6 (−43.8 to −41.5) | −1.2 (−1.3 to −1.2) |
| Day 810 | 2550 | −42.4 (−43.5 to −41.2) | −1.2 (−1.3 to −1.2) |
| Day 990 | 2431 | −42.6 (−43.8 to −41.4) | −1.2 (−1.3 to −1.2) |
| Day 1080 | 2377 | −49.7 (−50.9 to −48.6) | −1.4 (−1.5 to −1.4) |
| Day 1080/EOS | 2731 | −49.4 (−50.4 to −48.3) | −1.5 (−1.5 to −1.4) |
| **ASCVD population** | | | |
| Day 90 | 2662 | −47.6 (−48.7 to −46.5) | −1.3 (−1.3 to −1.3) |
| Day 270 | 2484 | −45.2 (−46.4 to −44.1) | −1.2 (−1.3 to −1.2) |
| Day 450 | 2391 | −43.9 (−45.1 to −42.6) | −1.2 (−1.2 to −1.2) |
| Day 630 | 2225 | −44.0 (−45.3 to −42.7) | −1.2 (−1.2 to −1.2) |
| Day 810 | 2125 | −43.9 (−45.1 to −42.6) | −1.2 (−1.2 to −1.2) |
| Day 990 | 2016 | −44.3 (−45.6 to −43.0) | −1.2 (−1.5 to −1.2) |
| Day 1080 | 1966 | −51.5 (−52.7 to −50.2) | −1.4 (−1.5 to −1.4) |
| Day 1080/EOS | 2205 | −51.0 (−52.2 to −49.9) | −1.4 (−1.4 to −1.4) |
| **ASCVD risk equivalent population** | | | |
| Day 90 | 562 | −37.1 (−39.4 to −34.8) | −1.4 (−1.5 to −1.3) |
| Day 270 | 528 | −34.3 (−37.0 to −31.5) | −1.3 (−1.5 to −1.2) |
| Day 450 | 496 | −34.7 (−37.3 to −32.1) | −1.4 (−1.5 to −1.2) |
| Day 630 | 436 | −35.5 (−38.1 to −32.9) | −1.4 (−1.5 to −1.3) |
| Day 810 | 425 | −34.9 (−37.7 to −32.1) | −1.4 (−1.5 to −1.3) |
| Day 990 | 415 | −34.4 (−37.4 to −31.5) | −1.4 (−1.5 to −1.2) |
| Day 1080 | 411 | −41.5 (−44.5 to −38.6) | −1.6 (−1.7 to −1.5) |
| Day 1080/EOS | 526 | −42.4 (−45.0 to −39.9) | −1.7 (−1.8 to −1.5) |

Percentage and absolute changes are evaluated from the parent study baseline.

ASCVD = atherosclerotic cardiovascular disease; CI = confidence interval; LDL = low-density lipoprotein; EOS = end of study; n = number of patients at each visit.

**Supplementary Table 5. Proportion of Patients Achieving LDL-C Goals for Their Level of ASCVD Risk Across ORION-9, ORION-10, and ORION-11, and ORION-8**

|  | **Phase 3 Inclisiran-Inclisiran Arm,**  **N = 1512,**  **n (%)** | **Phase 3 Placebo-Inclisiran Arm,**  **N = 1478,**  **n (%)** |
| --- | --- | --- |
| **ORION-9, ORION-10, and ORION-11** | | |
| Day 90 | 77.9% | 12.2% |
| Day 150 | 82.7% | 13.4% |
| Day 270 | 73.1% | 14.4% |
| Day 330 | 81.7% | 15.4% |
| Day 450 | 69.7% | 16.4% |
| Day 510 | 81.4% | 16.2% |
| Day 540 | 78.1% | 16.9% |
| **ORION-8** | | |
| Day 90 | 72.8% | 78.3% |
| Day 270 | 70.8% | 74.6% |
| Day 450 | 70.5% | 71.6% |
| Day 630 | 70.8% | 71.5% |
| Day 810 | 70.5% | 72.5% |
| Day 990 | 71.3% | 73.6% |
| Day 1080 | 78.7% | 79.6% |
| Day 1080/EOS | 78.2% | 79.1% |

ASCVD = atherosclerotic cardiovascular disease; LDL-C = low-density lipoprotein cholesterol; EOS = end of study.

**Supplementary Table 6. Treatment-Emergent Adverse Events at the Injection Site by Maximum Severity**

| **Maximum Severity of TEAE at Injection Site** | **Total N = 3274 n (%)** |
| --- | --- |
| **Mild** | 167 (5.1) |
| **Moderate** | 26 (0.8) |
| **Severe** | 0 |

N = total number of patients; n = number of patients in each category; TEAE = treatment‑emergent adverse event.

**Supplementary Table 7. Inclisiran-Associated ADAs**

|  | **ORION-8, Excluding ORION-1 Inclisiran Patients (N = 3061),**  **m/n (%)** |
| --- | --- |
| **Inclisiran-associated ADA*** | 162/2945 (5.5) |
| Persistent | 50/2945 (1.7) |
| Transient | 112/2945 (3.8) |
| **No inclisiran-associated ADA response** | 2783/2945 (94.5) |

The ADA response was determined by the pooled analysis of ORION-3, ORION-9, ORION-10, ORION-11, and ORION-8 ADA data. ORION-1 inclisiran arm patients were excluded from the analysis because the laboratory protocol used in the ORION-1 ADA assessment was different from that in other studies.

ADA = antidrug antibody; m = number of patients in each category; n = number of patients with negative baseline ADA and at least one post-baseline ADA assessment.

*Inclisiran-associated ADA: negative ADA sample at baseline and at least one positive ADA sample post-baseline.

# SUPPLEMENTARY FIGURE

**
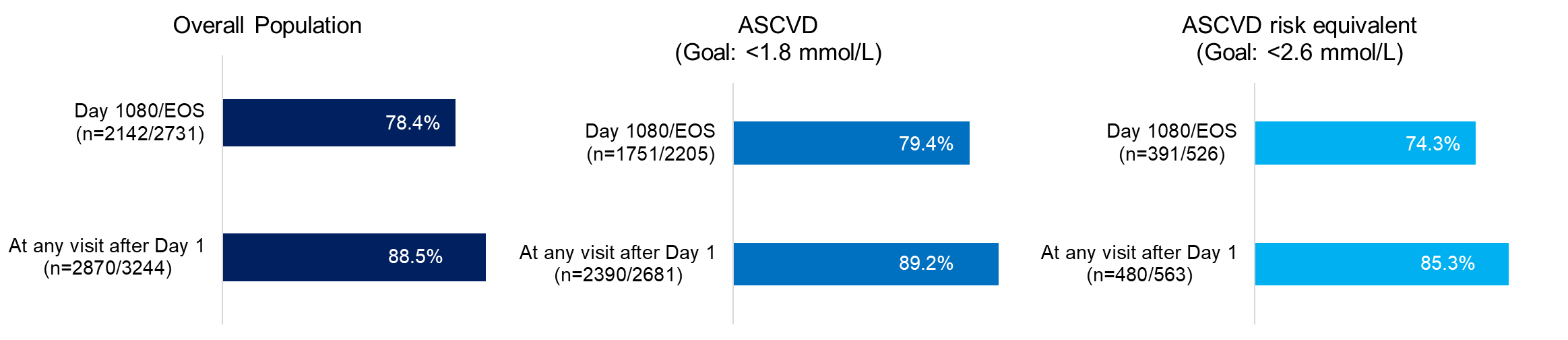
**

**Supplementary Figure 1. Proportion of Patients Achieving LDL-C Goals at Any Visit throughout ORION-8**

ASCVD = atherosclerotic cardiovascular disease; LDL-C = low-density lipoprotein cholesterol; EOS = end of study.
